# Assessment of midwifery care providers intrapartum care competencies, in four sub-Saharan countries: a mixed-method study protocol

**DOI:** 10.1186/s12978-021-01109-8

**Published:** 2021-02-27

**Authors:** Ann-Beth Moller, Joanne Welsh, Mechthild M. Gross, Max Petzold, Elizabeth Ayebare, Effie Chipeta, Hashim Hounkpatin, Bianca Kandeya, Beatrice Mwilike, Antoinette Sognonvi, Claudia Hanson

**Affiliations:** 1 School of Public Health and Community Medicine, Institute of Medicine, University of Gothenburg, Gothenburg, Sweden; 2 Midwifery Research and Education Unit, Hannover Medical School, Hannover, Germany; 3 Department of Nursing, Makerere University, Kampala, Uganda; 4 College of Medicine, The Centre for Reproductive Health, University of Malawi, Blantyre, Malawi; 5 Centre de Recherche en Reproduction Humaine et en Démographie (CERRHUD), Cotonou, Benin; 6 Muhimbili University of Health and Allied Sciences, Dar Es Salaam, Tanzania; 7 Global Public Health, Karolinska Institute, Stockholm, Sweden; 8 Department of Disease Control, London School of Hygiene and Tropical Medicine, London, UK

**Keywords:** Midwifery care providers, Intrapartum care, In-service training, Multi country study, Sub-Saharan Africa

## Abstract

**Background:**

We aim to assess competencies (knowledge, skills and attitudes) of midwifery care providers as well as their experiences and perceptions of in-service training in the four study countries; Benin, Malawi, Tanzania and Uganda as part of the Action Leveraging Evidence to Reduce perinatal mortality and morbidity in sub-Saharan Africa project (ALERT). While today more women in low- and middle-income countries give birth in health care facilities, reductions in maternal and neonatal mortality have been less than expected. This paradox may be explained by the standard and quality of intrapartum care provision which depends on several factors such as health workforce capacity and the readiness of the health system as well as access to care.

**Methods:**

Using an explanatory sequential mixed method design we will employ three methods (i) a survey will be conducted using self-administered questionnaires assessing knowledge, (ii) skills drills assessing basic intrapartum skills and attitudes, using an observation checklist and (iii) Focus Group Discussions (FGDs) to explore midwifery care providers’ experiences and perceptions of in-service training. All midwifery care providers in the study facilities are eligible to participate in the study. For the skills drills a stratified sample of midwifery care providers will be selected in each hospital according to the number of providers and, professional titles and purposive sampling will be used for the FGDs. Descriptive summary statistics from the survey and skills drills will be presented by country. Conventional content analysis will be employed for data analysis of the FGDs.

**Discussion:**

We envision comparative insight across hospitals and countries. The findings will be used to inform a targeted quality in-service training and quality improvement intervention related to provision of basic intrapartum care as part of the ALERT project.

*Trial registration:* PACTR202006793783148—June 17th, 2020.

## Plain abstract

While today more women in low- and middle-income countries give birth in health care facilities, reductions in maternal and neonatal mortality have been less than expected. This may partly be explained by the quality of care provision, which dependent on several factors, including appropriately trained midwifery care providers. This study is a component of the Action Leveraging Evidence to Reduce perinatal morTality and morbidity in sub-Saharan Africa (ALERT) project—a quality improvement and implementation science project. This paper outlines a study designed to assess the competencies (knowledge, skills and attitudes) of midwifery care providers in Benin, Malawi, Tanzania and Uganda. The study will use mixed methods to assess these aspects; a quantitative survey will assess the knowledge of midwifery care providers, observation of a skills drill session will assess midwifery care provider skills and attitudes, and Focus Group Discussions will be used to explore midwifery care providers’ experiences and perceptions of in-service training. Findings will be used to inform the development of an in-service training programme on basic intrapartum care for midwifery care providers.

## Background

In 2016, at the start of the Sustainable Development Goals (SDGs) era [1], pregnancy-related and newborn preventable morbidity and mortality remained unacceptably high. Despite reductions in maternal mortality between 2000 and 2017, every day approximately 810 women died in 2017 [2] and almost 6700 newborns died daily in 2019 [3]. Furthermore, estimates suggest that in 2019, for every 1000 total births, 13.9 babies were stillborn [4]. The majority of preventable maternal and newborn deaths occur in low- and middle-income countries, among the low-income population, in addition to those living in fragile settings.

Maternal and newborn deaths are a particularly sensitive indicator of the quality of a health system. Despite increases in the number of deaths from indirect causes such as non-communicable diseases, over 70% of deaths continue to occur from direct causes including haemorrhage, eclampsia, sepsis and abortion. More than 85% of newborn deaths are due to complications during and immediately after birth. High quality management of complications during this period can therefore have a significant impact on maternal and newborn deaths and stillbirths [5].

Evidence suggests that up to 2.5 million lives can be saved using evidence-based quality care [6]. Several global initiatives have been laid out such as the Sustainable Development Goals [7], the Global Strategy for Women’s, Children’s and Adolescents’ Health (2016–2030) [8], “Strategies toward ending preventable maternal mortality” (EPMM) [9], and “Every Newborn Action Plan” (ENAP) [10]. These global initiatives have set ambitious targets for maternal and newborn health outcomes which will require quality care, strong and effective strategies and commitments.

Quality of intrapartum care is paramount to ensure the optimal health outcomes for women and newborns. In this project we define quality of care based on the “The Quality Maternal and Newborn Care Framework” developed by Renfrew et al. [11] which describes the components and characteristics of quality care. The framework is structured around five components of care; Practice categories, Organisation of care, Values, Philosophy and Care providers. Within each of these components the characteristics of care for childbearing women and infants, as well as for childbearing women and infants with complications, are framed.

A critical progress indicator, explicitly adopted for SDG 3 (indicator 3.1.2) and by both the Global Strategy for Women’s, Children’s and Adolescents’ Health, 2016–2030, and EPMM, is the “proportion of births attended by skilled health personnel” (SBA). [1, 8, 12].

For universal SBA coverage and facility births to improve maternal and newborn health outcomes it is imperative that services provided follow evidence-based recommendations and are of high quality. [13, 14] However, the provision of quality intrapartum care is affected by several factors such as access, the health workforce capacity, an enabling environment and social and cultural determinants. Knowledge and skill deficits are not only related to specific emergency obstetric care functions but also basic intrapartum competencies. Accurate comprehension of competencies of midwifery care providers plus enhanced measurement and monitoring are all essential elements to better understand the paradox between expanded SBA coverage/facility birth and maternal and newborn health outcomes. Good quality midwifery care has the potential to reduce both maternal and newborn mortality and morbidity in high, low and lower- and middle-income countries [15], and it would be expected that maternal and neonatal mortality would decrease when midwifery care providers are educated to provide high level quality evidence based intrapartum care. Quality includes the respectful care of women. Disrespect and abuse of women during childbirth has been widely reported in low-and-middle income countries, including documented instances of physical abuse, non-consented clinical care, limited attention to confidentiality and dignity, discrimination, abandonment, and detention in facilities [16].

Several studies have evaluated in-service training programmes which focused on emergency obstetric care with their findings showing that the training did, to a varying degree, result in improvements in healthcare provider knowledge/skills and changes in clinical practice [17–19]. But improved health outcomes such as a reduction in still births, maternal mortality and the number of cases of post-partum haemorrhage are not necessarily reflected after these trainings. More recently, the COVID-19 pandemic has highlighted the need for in- service training specific to diagnosing and caring for patients with the virus, as well as in infection prevention and control.

Little in-service training has focused on basic midwifery competencies in relation to the importance of; the admission assessment, communication, respectful care, vaginal assessment and documentation [15].

Table 1 provides definitions of terminology used for this study.

**Table 1:** Definition of terminology used for this study

Term	Definition	Conceptual characteristics
Behaviour*	Observable conduct towards other people or activities that expresses a competency. Behaviours are durable, trainable and measurable	Observable attribute, often applied in combination, i.e. several behaviours may contribute towards one competency
Competency*	The observable ability of a person, integrating knowledge, skills, and attitudes in their performance of tasks. Competencies are durable, trainable and, through the expression of behaviours, measurable	Not time limited, i.e. durable through multiple activities Can develop/improve or erode over time
In-service training	Training that is given to employees during the course of their employment to update their professional knowledge, skills and competence	
Knowledge*	Understanding of, or information about a subject. The informational base of competence and skills	An unobservable attribute of competence inferred through performance or determined through testing
Midwifery care providers	Providers of midwifery care are competent maternal and newborn health professionals educated, trained and regulated to national and/or international standards. They provide skilled, evidence-based and compassionate care to women, newborns and families. Providers of midwifery care:	
	Promote and facilitate normal physiological, social and cultural processes throughout the childbearing continuum with a continuity of care philosophy	
	Seek to prevent and manage maternal and newborn complications	
	Consult and refer to other health services where required	
	Respect women’s individual circumstances and views, providing sensitive and dignified care	
Pre-service training	A formal learning programme which takes place prior to and as a prequisite for employment in a service setting	
Skill*	A specific cognitive or motor ability that is typically developed through training and practice	Observable (physical) and unobservable (cognitive) attribute, often applied in combination, i.e. several skills may contribute towards one competency and/or activity

*Source: Mills JA, Middleton JW, Schafer A, Fitzpatrick S, Short S, Cieza A. Proposing a re-conceptualisation of competency framework terminology for health: a scoping review. Hum Resour Health. 2020 Feb 21;18(1):15

## Study purpose and aims

This study is a component of the Action Leveraging Evidence to Reduce perinatal morTality and morbidity in sub-Saharan Africa project (ALERT)—“Positioning Midwifery” (https://ki.se/en/gph/the-alert-intervention-research-project). ALERT is a hospital maternity-based quality improvement and implementation science project in Benin, Malawi, Tanzania and Uganda [20], which aims to implement an evidence-based intervention and reduce in-facility perinatal mortality and morbidity.

This formative work aims to inform the development of an in-service training programme. We will conduct an assessment of the basic intrapartum competencies (knowledge, skills and attitudes) as well as an exploration of provider experiences and perceptions of in-service training among the midwifery care providers in the 16 ALERT project hospitals. An in-service training programme will be developed and contextualized based on requirements identified by end-users and on local needs.

### Study research questions

- What is the knowledge of midwifery care providers providing intrapartum care in the project hospitals?
- What are the skills and attitudes of midwifery care providers providing intrapartum care in the study hospitals?
- What experiences and perceptions are held by midwifery care providers in relation to in-service training that they have received?

## Methods

### Study design

The study will use a cross sectional sequential exploratory (QUAN—qual) mixed method design. The mixed-method paradigm is based on the principles and logic of pragmatism. According to this paradigm, a mixed use of qualitative and quantitative approaches results in a better understanding of the problem [21]. In this case, a mixed-method design was chosen due to the complexity of the research questions.

Employing a participatory approach is anticipated to help providers in acknowledging possible gaps in their intrapartum competencies. One arm of the ALERT project is end-user participation. This enables providers to voice their training needs and allows the intervention to develop in a manner that meets these needs. This will be achieved by using findings from our baseline qualitative and quantitative data that includes the opinions and views of both health care providers and clients regarding the gaps in intrapartum care. While training is likely to be successful if the providers understand why the topic is important to address, ownership of the whole process and responsibility for their own learning are elements which will guide the training. Applying these participatory concepts is expected to enhance engagement during the in-service training.

The quantitative study will provide an assessment of knowledge, skills and attitude of the midwifery care providers related to basic intrapartum care. The FGDs will enable provider’s experiences and perceptions of in-service training to be explored and contextualised from multiple perspectives.

This protocol is conducted in accordance with the STROBE Statement—for cross-sectional studies [22] included in Additional file 1.

**Fig. 1:**
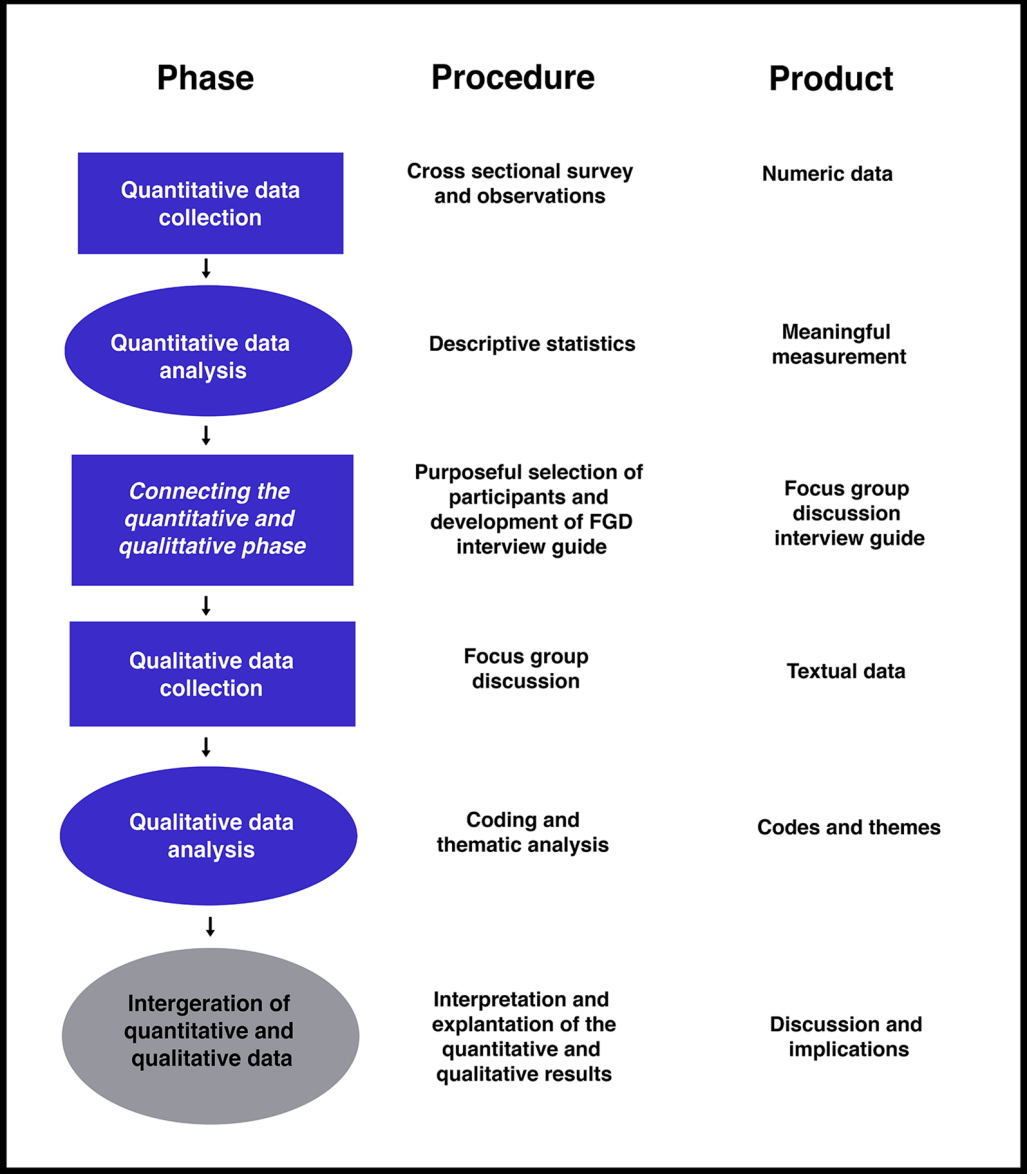
Methodological approach of the assessment of intrapartum competencies and experiences and perceptions related to in-service training

The research approach is summarised in Fig. 1.

### Study setting

The study will be conducted in maternity wards of all the 16 ALERT project hospitals in Benin, Malawi, Tanzania and Uganda. The hospitals were selected based on the following criteria:

i. Minimum caseload of 2500 births per year;
ii. Caesarean section and blood transfusion services available;
iii. Preferably located in rural areas, and
iv. Consist of a mix of typical public but also private-not-for-profit (faith-based) hospitals to represent the typical landscape of hospitals providing intrapartum care.

The number of midwifery care providers in each hospital ranges from 20 to 70 providers.

### Target population and sampling

For the survey which aims to assess knowledge, all midwifery care providers providing intrapartum care in the 16 maternity wards are invited to participate. For the skills drills assessment, a stratified sample of midwifery care providers will be randomly selected from each hospital/maternity ward according to the size of the facility and ensuring that all  professional titles; midwives, nurse-midwives, doctors etc. are represented proportionately.

We will apply a stratified sample stratifying by provider professional titles and as described in Table 2. We will sample 25–30% of the providers in each hospital depending on the number of providers working at each hospital.

**Table 2:** Midwifery care provider selection details

Number of midwifery care providers at the facility/maternity ward	Number of providers providing intrapartum care to be randomly selected
≤ 20	5 (25%)
21–30	7–10 (25–30%)
31–70	10–20 (around 30%)

Two FGDs will be conducted in each study country and a "purposive" sample will be used. Six to −10 providers will be invited to participate based on their function at the maternity ward. Focus groups will be homogenous in composition in terms of sex, age and hierarchy, to support participants to feel more comfortable expressing their opinions.

### Study tools

The quantitative part of the study will apply two different data collection tools; a self-administered survey and an observation skills drills checklist. The assessment will focus on basic intrapartum care as described in the Essential Competencies for Midwifery Practice Framework 2019 [23] as several training packages and initiatives have focused on emergency intrapartum care training [24, 25] and often basic midwifery care has not been given the appropriate and needed attention.

The International Confederation of Midwives (ICM) Essential Competencies for Midwifery Practice Framework, 2019 update [23] was used as the theoretical framework for the development of the self-administered survey and the observation checklist related to the skills drills. Details of the Essential Competencies for Midwifery Practice Framework 2019 related to intrapartum care is described in Additional file 2.

The self-administered survey consists of seven sections illustrated in Box 1.

The questions are mainly closed questions with a mix of multiple choice and some “yes, no or don’t know” questions.

The full survey is included in Additional file 3.

The observation study will be carried out as skills drills due to ethical considerations. The observation study has two components related to two clinical scenarios. The first scenario will assess the skills and attitudes of midwifery care providers during the admission process. The second scenario will focus on the second stage of labour and immediate postpartum newborn care. For the skills drills a “Laerdal Mama Birthie Kit” will be used [26]. One ALERT team member will play the “woman” (the client) and one the companion (client’s companion) and will be given instructions on how to answer the questions that the midwifery care provider may ask using the “drill script”—[see Additional file 4].

For the skills drills a checklist will be used to record which clinical practices are performed and which may not be executed. The checklist will include all clinical evidence-based aspects which would be expected to be addressed in each scenario.

The complete skills drills checklist tool is included in Additional file 5.

The tools and skills drills scripts will be translated into French and Swahili.

For the FGDs an interview guide will be used. The questions in the interview guide will aim to encourage participants to discuss their experiences and perceptions of in-service training and will contain probe, follow-up and exit questions. Discussions during the course of the FGDs may prompt further questions from the FGD facilitator and the participants.

### Pre-test

The pre-test study facilities will be selected to be representative of at least one of the study hospitals in terms of level of facility type and size of workforce.

A pre-test of the two tools will be conducted. This will allow understanding of the content of the survey to be assessed, any language problems to be identified, and allow for an approximation of how much time it takes to complete the survey. Pre-testing of the skills drills observations checklist as well as the script for the client will also take place. Feedback will be used to modify the tools if needed.

### Data collection

The data collection will be conducted by one ALERT co-investigator with a midwifery background and a data collection assistant with nurse-midwifery training in each project country. The teams will be trained by an ALERT team member in conducting both the quantitative and qualitative components of the study.

Tablet computers will be used for data collection. The providers will fill in the survey in an undisturbed location and the survey will be conducted at different time points to ensure providers working different shifts will be able to complete the survey during working hours.

For the skill drills the data collectors will use an electronic observational checklist to record the clinical care provided by the study participants.

The FGDs will take place after the quantitative study, and at least one to two focus groups will be conducted in each of the facilities. Each FGD is expected to last between 60 and 90 min and will take place in meeting rooms in the hospitals.

We will ask for participant consent to record but cannot guarantee that we will be allowed to record. If not, there will be two notetakers in each group. Notes will be transferred to a NVivo software programme for analysis.

The qualitative team members in each country will do the coding and be involved in the development of the code book.

Assuming there will be discrepancies in coding they will dealt with through continuous discussions on interpretation of data among the team members in each country and in a second phase across countries. The interpretation of data and the appropriate codes will also be assessed through method and participant triangulation.

### Data management and analysis

Data from the surveys and skills drills observation tools will be exported from the tablets to REDCap (Research Electronic Data Capture). The data will be stored on a server at Karolinska Institute, Stockholm, Sweden. The collected data will be analysed using STATA and descriptive statistical methods and will generate summary statistics for each hospital and aggregated at country level. Data collected from the survey will be analysed using a summary score from each sub-section. The findings from the observation study will be quantified and a final score will be given to the two different skills drills scenarios. The findings from the quantitative studies will be used to triangulate and understand the potential (dis)connect between competencies and the actual clinical practices, and the perceptions and experiences related to in-service training.

Any comments provided on the survey and/or the observation tools will also be analysed.

All FGDs data will be transcribed in the local languages verbatim (English, French and Swahili), then where required, translated into English and transferred into electronic files containing one transcript for each data collection event.

The data from the FGDs will be coded and analysed using “thematic analysis” [27]. Thematic analysis allows for patterns (themes) arising from the data to be identified, analysed and reported in a systematic way. The analysis will use an inductive, exploratory approach rather than a confirmatory approach driven by specific questions and ideas. NVivo will be used to support the management and coding of data collected in FGDs.

Data from the different assessments will be combined to facilitate the interpretation and understanding of midwifery care providers’ experiences and perceptions of in-service training and how this impacts their own clinic practice. A joint display will be developed to provide a structure to discuss the integrated analysis and assist both researchers and readers in understanding how this study may provide new insights [28].

### Ethical considerations

Study participants will be asked to provide informed, written consent prior to participation in this study and will, at any given time, be able to withdraw from the study.

Box 1 Content of the self-administered survey sections Section 1. Provider characteristics (personnel information, educational background and general information related to current job);

Section 2. Working environment (history of in-service training);

Section 3. Triage and referral;

Section 4. First stage management of labour;

Section 5. Second stage management of labour;

Section 6. Third stage management of labour; and.

Section 7. Reporting and documentation, and handover between shifts.

The survey includes three questions specifically related to the COVID pandemic.

## Discussion

This study will provide a comprehensive and comparative analysis of competencies of midwifery care providers as well as their experiences and perceptions of in-service training. These findings will inform the development of an end-user focused in-service training programme to be implemented in all the ALERT project hospitals. Employing a participatory approach is anticipated to help providers in acknowledging possible gaps in their intrapartum competencies. The participatory approach will be centred on the providers and developed according to their needs. While training is likely to be successful if the providers understand why the topic is important to address, ownership of the whole process and responsibility for their own learning  are elements which will guide the training. Applying these participatory concepts is expected to enhance engagement during the in-service training.

The study also has some limitations as it will not be possible to assess all aspects of basic intrapartum care knowledge in the survey, but questions aim to cover a wide range topics related to basic care. Owing to diversity among the study settings and social determinants related to childbirth, the local health workforce as well as cultural aspects it may not be possible to generalize study findings, but transferability of results may be applicable. Due to ethical reasons skills drills were selected in place of conducting direct observations which may not provide as much insight into practices and behaviours. Nevertheless, observational studies participants have the tendency to change their behaviours simply as a result of being observed—the phenomenon known as the Hawthorne effect [29].

For the FGDs convenience sampling will be used to invite providers based on their accessibility and willingness to participate. This leads to “volunteer bias” whereby those taking part in the FGDs may not accurately represent the general population. On the other hand, purposive sampling is widely recommended since focus group discussion relies on the ability and capacity of participants to provide relevant information. FGDs lend themselves to biases commonly encountered in any group setting such as dominance effect (a dominant individual shape the discussion), halo effect (the perceived status of a group member influences the discussion), groupthink (the members in a group tend to think similarly to maintain group cohesion). [30] Even though biases may affect the outcome, the information from these discussions may provide granularities on important information which can’t be captured in the survey and the skills drills assessment.

We have received administrative clearances and support from all participating hospitals during the proposal development and approval process. We therefore believe that this support will continue even during the intervention period of the study.

We anticipate that the experiences and results in some of the sites will encourage to upscale services in other sites (e.g. including a companion during labour). One of the main advantages of ALERT is not only that study sites will be able to discuss their experiences and results but that these experiences and results can be discussed and compared across the ALERT countries.

By inviting providers to reflect on the results of the competency assessment we aim to gain an understanding of their priority training needs. By focusing on these needs as we develop the in-service training package, we aim to improve competency, which in turn, will contribute to an improvement in quality of care provided.

The study findings may contribute to informing the global agenda and national policies related to improvement in the provision of intrapartum care and the understanding of inconsistencies between the increased number of women given birth in facilities and the current level of maternal and neonatal mortality [31, 32].

## Supplementary Information

**Additional file 1.** STROBE statement.

**Additional file 2.** The ICM Essential Competencies for Midwifery Practice Framework.

**Additional file 3.** Self-administered questionnaire.

**Additional file 4.** Skills drills script.

**Additional file 5:** Skills drills checklist.

## Data Availability

Not applicable.

## Abbreviations

ALERT: Action Leveraging Evidence to Reduce perinatal morTality and morbidity in sub-Saharan Africa
ENAP: Every newborn action plan
EPMM: Strategies toward ending preventable maternal mortality
FGD: Focus group discussion
ICM: The International Confederation of Midwives
REDCap: Research Electronic Data Capture
SBA: Skilled birth attendant
SDGs: Sustainable development goals
