# Supplementary material for: Assessment of midwifery care providers intrapartum care competencies, in four sub-Saharan countries: a mixed-method study protocol
Source: Reprod Health. 2021 Feb 27;18:50. doi: 10.1186/s12978-021-01109-8 (PMC7912468; doi:10.1186/s12978-021-01109-8)
Supplement: Supplementary file 2 — Additional file 2. The ICM Essential Competencies for Midwifery Practice Framework. [file 12978_2021_1109_MOESM2_ESM.pdf]

Additional file 2.

The ICM Essential Competencies for Midwifery Practice Framework – Topic related to care during labour and childbirth.

Competencies in this category are about assessment and care of women during labour that facilitates physiological processes and a safe birth, the immediate care of the newborn infant, detection of complications in mother or infant, stabilisation of emergencies, and referral as needed.

Source: The International Confederation of Midwives (ICM) Essential Competencies for Midwifery Practice, 2019 update. [Available from: https://www.internationalmidwives.org/our-work/policy-and-practice/essential-competencies-for-midwifery-practice.html].

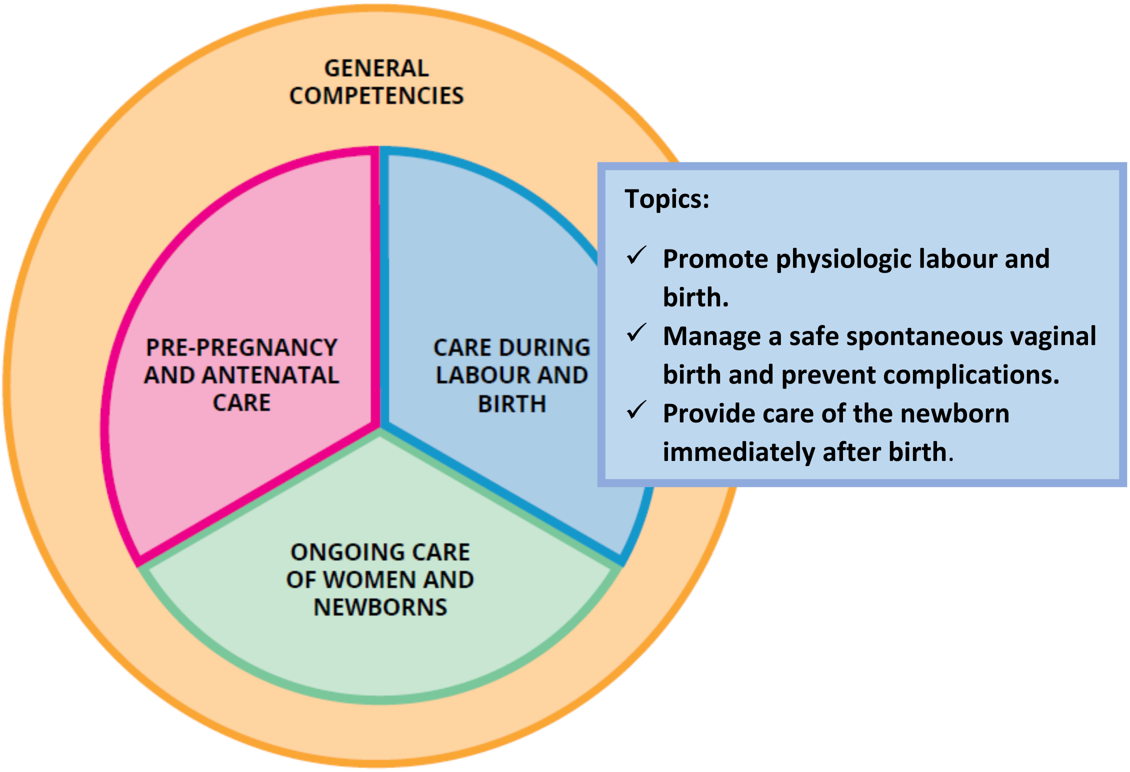

Knowledge, skills and behaviours according to ICM Essential Competencies

<table>
<tr><td colspan="2">Subject/topics
Promote physiologic labour and birth</td></tr>
<tr><td>Knowledge</td><td>Skills and behaviours</td></tr>
<tr><td>Anatomy of maternal pelvis and fetus; mechanisms of labour for different fetal presentations</td><td rowspan="6">• Provide care for a woman in the birth setting of her choice, following policies and protocols
• Obtain relevant obstetric and medical history
• Perform and interpret focused physical examination of the woman and fetus
• Order and interpret laboratory tests if needed
• Assess woman’s physical and behavioural responses to labour
• Provide information, support, and encouragement to woman and support persons throughout labour and birth
• Provide respectful one-to-one care
• Encourage freedom of movement and upright positions
• Provide nourishment and fluids
• Offer and support woman to use strategies for coping with labour pain, e.g. controlled breathing, water immersion, relaxation, massage, and pharmacologic modalities when needed
• Assess regularly parameters of maternal-fetal status, and e.g. vital signs, contractions, cervical changes, and fetal descent
• Use labour progress graphic display to record findings and assist in detecting labour delay or other complications
• Augment uterine contractility judiciously using non-pharmacological or pharmacological agents to prevent non-progressive labour
• Prevent unnecessary routine interventions, e.g. amniotomy, electronic fetal monitoring, directed closed glottis pushing</td></tr>
<tr><td>Physiologic onset and progression of labour</td></tr>
<tr><td>Evidence about interventions in normal labour and birth</td></tr>
<tr><td>Cultural and social beliefs and traditions about birth</td></tr>
<tr><td>Signs and behaviours of labour progress; factors that impede labour progress</td></tr>
<tr><td>Methods of assessing fetus during labour</td></tr>
</table>

Knowledge, skills and behaviours according to ICM Essential Competencies

<table>
<tr><td colspan="2">Subject/topics
Manage a safe spontaneous vaginal birth and prevent complications</td></tr>
<tr><td>Knowledge</td><td>Skills and behaviours</td></tr>
<tr><td>Methods of conducting birth to protect perineal integrity</td><td rowspan="5">• Support the woman to give birth in her position of choice
• Ensure presence of clean necessary supplies and source of warmth
• Coach woman about pushing to control expulsion of presenting part, avoid routine episiotomy
• Undertake appropriate maneuvers and use maternal position to facilitate vertex, face, or breech birth
• Delay cord clamping
• Manage nuchal cord
• Assess immediate condition of newborn
• Provide skin to skin contact and warm environment
• Deliver placenta and membranes and inspect for completeness
• Assess uterine tone, maintain firm contraction, and estimate and record maternal blood loss
• Inspect vaginal and perineal areas for trauma, and repair as needed, following policies and protocols</td></tr>
<tr><td>Evidence about conduct of third stage, including use of uterotonics</td></tr>
<tr><td>Potential complications and their immediate treatment e.g. shoulder dystocia, and excessive bleeding</td></tr>
<tr><td>Signs of placental separation; appearance of normal placenta, membranes, and umbilical cord</td></tr>
<tr><td>Types of perineal and vaginal trauma requiring repair and suturing techniques</td></tr>
</table>

Knowledge, skills and behaviours according to ICM Essential Competencies

<table>
<tr><td colspan="2">Subject/topics
Provide care of the newborn immediately after birth</td></tr>
<tr><td>Knowledge</td><td>Skills and behaviours</td></tr>
<tr><td>Normal transition to extra-uterine environment</td><td rowspan="6">• Use standardized method to assess newborn condition in the first minutes of life (Apgar or other); refer if needed
• Institute actions to support breathing and oxygenation
• Provide a safe warm environment for initiating breastfeeding and attachment (bonding) in the first hour of life
• Conduct a complete physical examination of newborn in presence of mother/family; explain findings and expected changes e.g. colour of extremities, moulding of head. Refer for abnormal findings.
• Institute newborn prophylaxis e.g. ophthalmic infection, and hemorrhagic disease, according to policies and guidelines
• Promote care by mother, frequent feeding and close observation
• Involve partner/support persons in providing newborn care</td></tr>
<tr><td>Scoring systems to assess newborn status</td></tr>
<tr><td>Signs indicating need for immediate actions to assist transition</td></tr>
<tr><td>Appearance and behaviour of healthy newborn infant</td></tr>
<tr><td>Method of assessing gestational age of newborn infant</td></tr>
<tr><td>Needs of small for gestational age and low birth weight infants</td></tr>
</table>
