# Supplementary material for: Assessment of midwifery care providers intrapartum care competencies, in four sub-Saharan countries: a mixed-method study protocol
Source: Reprod Health. 2021 Feb 27;18:50. doi: 10.1186/s12978-021-01109-8 (PMC7912468; doi:10.1186/s12978-021-01109-8)
Supplement: Supplementary file 3 — Additional file 3. Self-administered questionnaire. [file 12978_2021_1109_MOESM3_ESM.pdf]

Unique identifier for health facilities

- Uganda: UG (hospital; UG/01, UG/02, UG/03, UG/04)
- Malawi: MW (hospital; MW/01, MW/02, MW/03, MW/04)
- Benin: BJ (hospital; BJ/01, BJ/02, BJ/03, BJ/04)
- Tanzania: TZ (hospital; TZ/01, TZ/02, TZ/03, TZ/04)

Unique identifier for health care providers: H for any health care provider

Tool WP3: 3/1 (part 1 of 2)

Country	□□
Hospital	□□
Tool	□/□
Type	H
Participant	####

/ / / /H/##

Supervisor: Name (capital letters) ___________________
Signature_____________________
Date: ___/___/

We would like to ask you some questions about your education, workplace, skills, knowledge and competencies related to the care you provide to women in labour and childbirth in your hospital. The information will be used to inform the content of the in-service training programme which will be conducted in your hospital as part of the ALERT project.

Please follow the instruction indicated under each question.

Date (dd/mm/year/): ___/___/______

Section 1. Provider characteristics (personnel information, educational background and general information related to current job)

No	Variable name	Variable/Questions	Values/Answer option	Instructions	Comment
1	s1q1	What is your sex? (please tick one answer)	1. Male<br>2. Female		
2	s1q2_B	What is your highest school education? (please tick one answer)	1. Completed primary school<br>2. Completed ordinary secondary school<br>3. Completed advanced secondary school		
	s1q2_M	What is your highest school education? (please tick one answer	1. Completed primary school<br>2. Completed ordinary secondary school<br>3. Completed advanced secondary school		
	s1q2_T	What is your highest school education? (please tick one answer	1. Completed primary school<br>2. Completed ordinary secondary school<br>3. Completed advanced secondary school		
	s1q2_U	What is your highest school education? (please tick one answer	1. Completed primary school<br>2. Completed ordinary secondary school<br>3. Completed advanced secondary school		

No	Variable name	Variable/Questions	Values/Answer option	Instructions	Comment
3	s1q3_B	What is your highest degree? (please tick)	1. Certificate<br>2. Bachelor<br>3. Master<br>4. Doctoral degree		
	s1q3_M	What is your highest degree? (please tick)	1. Certificate<br>2. Diploma<br>3. Bachelor<br>4. Master<br>5. Doctoral degree		
	s1q3_T	What is your highest degree? (please tick)	1. Certificate<br>2. Diploma<br>3. Bachelor<br>4. Master<br>5. Doctoral degree		
	s1q3_U	What is your highest degree? (please tick)	1. Certificate<br>2. Diploma<br>3. Bachelor<br>4. Master<br>5. Doctoral degree		
4	s1q4	How many months/years were you in total in pre-service training in nursing/midwifery/clinical science? (please write)	1. Less than 2 years<br>2. More than 2 years		**Please indicate months if less than 2 years, otherwise entre years.**
5	s1q5	How many months of pre-service training did you receive specifically in midwifery care? (please write)	1. Please write number of months:		
6	s1q6_B	What is your professional title? (please tick one answer)	1. Nursing auxillary<br>2. Midwife<br>3. Nurse<br>4. Doctor<br>5. Other		**If other, please write.**
	s1q6_M	What is your professional title? (please tick one answer)	1. Maid<br>2. Midwife<br>3. Nurse<br>4. Doctor<br>5. Other		**If other, please write.**
	s1q6_T	What is your professional title? (please tick one answer)	1. Midwife<br>2. Nurse<br>3. Doctor<br>4. Other		**If other, please write.**

No	Variable name	Variable/Questions	Values/Answer option	Instructions	Comment
	s1q6_U	What is your professional title? (please tick one answer)	1. Midwife<br>2. Nurse<br>3. Doctor<br>4. Other		**If other, please write.**
7	s1q7_B	What is your title in your current job? (cadre name) (please tick one answer)	1. Registered Midwife<br>2. Registered Nurse<br>3.Doctor<br>4. Other		**If other, please write.**
	s1q7_M	What is your title in your current job? (cadre name) (please tick one answer)	1. Maid<br>2. Nurse-midwife technician<br>3. Registered nurse-midwife<br>4. Nurse Officer<br>5. Intern Clinical Technician<br>6. Clinical Technician<br>7. Clinical Officer<br>8. Medical Officer<br>9. Senior Medical Officer<br>10. Health Surveillance Assistant<br>11. Other		**If other, please write.**
	s1q7_T	What is your title in your current job? (cadre name) (please tick one answer)	1. Enrolled nurse-midwife<br>2. Registered nurse-midwife<br>3. Medical Assistant<br>4. Assistant Medical Officer<br>5. Medical Officer<br>6. Other		**If other, please write.**
	s1q7_U	What is your title in your current job? (cadre name) (please tick one answer)	1. Enrolled midwife<br>2. Registered midwife<br>3. Intern Nurse<br>4. Intern Doctor<br>5. Clinical Officer<br>6. Medical Officer<br>7. Obstetrician		**If other, please write.**

No	Variable name	Variable/Questions	Values/Answer option	Instructions	Comment
			8. Gynaecologist<br>9. Other		
8	s1q8	How many year(s) of experience do you have in providing care to women giving birth? (please indicate number year(s)) (please write)	Please write the number of whole year(s) experience you have:<br>(Please exclude the periods you have been on maternity/paternity leave or long-term sick leave).		
9	s1q9	What kind of shift do you typically do? (please tick)	1. Day<br>2. Evening<br>3. Night<br>4. Rotating shifts		
10	s1q10	Approximately, how many births did you conduct in the last month? (please write)	1. Please write the number of births:		

Section 2. Working environment (in-service training)

No	Variable Name	Variable/Questions	Values/Answer option	Instructions	Comment
1	s2q1	Are you registered with a professional association/organization? (please tick)	1. Yes<br>2. No<br>3. I don't know		If yes, please write which organization:
2	s2q2	Do you receive any supervision for your work	1. Yes<br>2. No<br>3. Sometimes		If yes, please describe

No	Variable Name	Variable/Questions	Values/Answer option	Instructions	Comment
		related to labour and childbirth? (please tick)			how the supervision is provided :
3	s2q3	Do you have access to resources for staff education and training?	1. Yes<br>2. No<br>3. Sometimes		
4	s2q4	Do you have access to resources to support you in caring for labouring women?	1. Yes<br>2. No<br>3. Sometimes		
5	s2q5	Are you informed about changes in your hospital that will affect your practice?	1. Yes<br>2. No<br>3. Sometimes		
6	s2q6	Do you feel supported by your manager?	1. Always<br>2. Sometimes<br>3. Don't know<br>4. Rarely<br>5. Never		
7	s2q7	Have you had any training on how to recognise or assess symptoms of COVID-19 among women?	1. Yes<br>2. No		
8	s2q8	Have you had any training on caring for a woman who is suspected of or has COVID-19?	1. Yes<br>2. No		
9	s2q9	Is personal protective equipment available to you in sufficient quantity to change between patients?	1. Yes<br>2. No<br>3. Sometimes		

Section 3. Triage and referral

No	Variable name	Variable/Questions	Values/Answer option	Instructions	Comment
1	s3q1	In your facility is there a written protocol in place for triage related to labour and birth? (please tick)	1. Yes<br>2. No<br>3. I don't know		
2	s3q2	Does your facility have a written protocol in place for referral during labour and birth? Only for non-referral hospitals (please tick)	1. Yes<br>2. No<br>3. I don't know<br>4. I work in a referral hospital		
3	s3q3	Does your facility have a written protocol in place for the assessment of women with symptoms of COVID-19?	1. Yes<br>2. No<br>3. I don't know		

Section 4. First stage management of labour

No	Variable name	Variable/Questions	Values/Answer option	Instructions	Comment
1	s4q1	If it is available, do you always use a partograph? (please tick)	1. Yes<br>2. No		
2	s4q2	Concerning eating and drinking, what do you typically recommend? (please tick)	1. Encourage to drink and eat as wanted.<br>2. Restrict food but encourage to drink.<br>3. Encourage food but restrict drink<br>4. Restrict both to drink and eat.<br>5. Abstain fully from drinking and eating.		
3	s4q3	How do you typically manage companionship, which is that the women in labour and childbirth has someone she knows with her and supporting her? (please tick)	1. Encourage strongly throughout labour and childbirth (1st and 2nd stage).<br>2. Encourage during labour (1st stage) but discourage during birth (2nd stage).<br>3. Discourage during labour (1st stage) but encourage during birth (2nd stage)		

No	Variable name	Variable/Questions	Values/Answer option	Instructions	Comment
			4. Do not encourage as not important according to my view.<br>5. Do not encourage as not policy at this facility.		
4	s4q4	According to what you were taught, how often should the fetal heart rate be checked and recorded during active phase of the 1st stage of labour? (please tick one answer)	1. Every 15 minutes<br>2. Every 20 minutes<br>3. Every 30 minutes<br>4. Every 60 minutes<br>5. I don't monitor<br>6. I don't know		

Section 5. Second stage management of labour

No	Variable name	Variable/Questions	Values/Answer option	Instructions	Comment
1	s5q1	According to what you were taught, how often should the fetal heart rate be monitored in the 2nd stage? (please tick one answer)	1. Every 5 minutes<br>2. Every 15 minutes<br>3. Every 30 minutes<br>4. Every 60 minutes<br>5. I don't monitor<br>6. I don't know		
2	s5q2	According to what you were taught, what are the benefits of giving birth while standing, squatting, or kneeling? (please tick all answers that apply)	1. Shorter second stage.<br>2. Less blood loss.<br>3. Lower risk of second-degree tears.<br>4. I did not learn about a benefit.		
3	s5q3	According to what you were taught, when do you decide to intervene during the second stage of labour? (please tick all that apply)	1. When the second stage extends beyond the standard duration.<br>2. When there is fetal distress.<br>3. When there is evidence of progress in the descent of the fetal head.		

No	Variable name	Variable/Questions	Values/Answer option	Instructions	Comment
4	s5q4	Were you taught in your pre-service training how to perform an episiotomy? (please tick)	1. Yes<br>2. No		
5	s5q5	Do you use/apply anaesthesia for suturing an episiotomy?	1. Yes<br>2. No		
6	s5q6	Immediate care for a normal newborn includes which of the following actions? (please tick all that apply)	1. Stimulating the baby by slapping the soles of the baby's feet.<br>2. Drying the baby.<br>3. Placing the baby in a baby warmer<br>4. Placing the baby skin-to-skin with the mother.		
7	s5q7	Under most circumstances, when should a woman be supported to begin breastfeeding? (please tick one item)	1. After the baby's first bath.<br>2. When the baby first starts to cry.<br>3. As soon as possible when the baby is ready within the first hour after birth.<br>4. When her milk comes in.		

Section 6. Third stage management of labour

No	Variable Name	Variable/Questions	Values/Answer option	Instructions	Comments
1	s6q1	How often should you monitor a woman (uterine tone, bleeding, BP, and pulse) in the	1. Every 5 minutes<br>2. Every 15 minutes<br>3. Every 30 minutes		

No	Variable Name	Variable/Questions	Values/Answer option	Instructions	Comments
		first two hours after giving birth? (please tick one answer)			
2	s6q2	In your daily work, how do you monitor blood loss during the third stage? (please tick all that apply)	1. Visual estimation of blood loss (e.g. counting the number of saturated pads).<br>2. Pulse rate and blood pressure measurement.<br>3. Hematocrit (red blood cell count).<br>4. I compare to other births where I have provided care.<br>5. Other		
3	s6q3	What are the common signs and symptoms of postpartum preeclampsia (please tick all that apply)?	1. High blood pressure, usually over 140/90.<br>2. High levels of protein in the urine.<br>3. Cold hands and feet.		
4	s6q4	If the baby is crying and does not need resuscitation, when should you clamp or tie the umbilical cord? (please tick one answer)	1. Immediately after birth.<br>2. 1 to 3 minutes after birth.<br>3. 5 minutes after birth.		
5	s6q5	What does APGAR stand for? (please write what each letter stands for)	1. A:<br>2. P:<br>3. G:<br>4. A:<br>5. R:		

Section 7. Reporting and documentation, and handover between shifts

No	Variable Name	Variable/Questions	Values/Answer option	Instructions	Comments
1	s7q1	Why do you think you need to document and report information about the women and their newborns as well as the care you provided?<br>(please tick all that apply)	1. The health managers need the data regularly.<br>2. My colleagues need to know about the women.<br>3. Data are used for quality improvement.<br>4. The women need the information.		
2	s7q2	For reporting and documentation which format are you using? (please tick)	1. Paper format (book).<br>2. Electronic format (computer).<br>3. Both paper and electronic format.		
3	s7q3	How much time is allocated to handover between shifts? (please indicate approximate minutes)	1. Minutes: _ _<br>2. I don't know		
