# Supplementary material for: Assessment of midwifery care providers intrapartum care competencies, in four sub-Saharan countries: a mixed-method study protocol
Source: Reprod Health. 2021 Feb 27;18:50. doi: 10.1186/s12978-021-01109-8 (PMC7912468; doi:10.1186/s12978-021-01109-8)
Supplement: Supplementary file 4 — Additional file 4. Skills drills script. [file 12978_2021_1109_MOESM4_ESM.pdf]

Skills drill script

Section 1: Admission

Vaginal bleeding	None
Loss of fluid from vagina	Yes – started losing fluid approximately one hour ago (If the provider asks it is clear and has no offensive smell)
Convulsions	None
Severe headache or blurred vision	None
Severe abdominal pain	None – only labour like pains
Respiratory difficulty	None
Fever	None

Section 3: Clinical history

Age	28
**Obstetric history**	
Number of pregnancies	This is my 3rd pregnancy
Number of abortions	None
Number of normal births	Two
Number of caesarean sections	None
Number of children born alive and still alive	Two
Number of stillbirths	None
Any maternal complication in previous pregnancy	None
Date and outcome of last pregnancy	24.05.2018 SVD, livebirth no maternal or neonatal complications
**General medical problems**	
Any medication	None
Use of alternative medication or herbs	No
Receiving treatment for TB/HIV	No – HIV Negative
**Gestational Age**	
Last menstrual period (LMP)	07.09.2019
Expected date of delivery	14.06.2020 (Gest is 38+2)
When did painful regular contractions start?	Contractions started 5 hours ago, but they have only been regular for 2 hours
Frequency and strength of contractions	3 contractions in 10 minutes, lasting 50 seconds
**Waters/membranes rupturing**	
When waters broke	One hour ago

What colour was the water	Clear
What smell did the waters have	None
Are you feeling baby's movements	Yes – movements are in a normal pattern

Section 4: Physical examination

Temperature	36.8C
Blood pressure	122/76
Pulse	81
Respiratory rate	18
Oedema	Mild oedema in the feet and ankles
Conjunctiva	Normal – no signs of anaemia

Section 5: Obstetric examination

Abdominal appearance	NAD – no scars noted
Fundal height<br>Fetal lie<br>Presentation<br>Engagement	38cm<br>Longitudinal<br>Cephalic<br>$2/5^{th}$ palpable
Contractions	3:10 minutes, 50 seconds long, moderate to strong on palpation, good resting tone between
Auscultates FH	138bpm

Section 6: Vaginal examination

Length<br>Texture<br>Dilation	<1cm long (fully effaced)<br>Soft and stretchy<br>8cm
Assess for membranes<br><br>Check for cord prolapse	Ruptured and clear liquor draining<br>No evidence of cord prolapse
Level of the presenting part	At spines -1
Fetal presentation<br>Fetal position	Cephalic<br>LOA
