# Supplementary material for: Assessment of midwifery care providers intrapartum care competencies, in four sub-Saharan countries: a mixed-method study protocol
Source: Reprod Health. 2021 Feb 27;18:50. doi: 10.1186/s12978-021-01109-8 (PMC7912468; doi:10.1186/s12978-021-01109-8)
Supplement: Supplementary file 5 — Additional file 5: Skills drills checklist. [file 12978_2021_1109_MOESM5_ESM.pdf]

Checklists to observe admission and childbirth related to the roleplays (WP3_Tool 3/2)

Unique identifier for health facilities

- Uganda: UG (hospital; UG/01, UG/02, UG/03, UG/04)
- Malawi: MW (hospital; MW/01, MW/02, MW/03, MW/04)
- Benin: BJ (hospital; BJ/01, BJ/02, BJ/03, BJ/04)
- Tanzania: TZ (hospital; TZ/01, TZ/02, TZ/03, TZ/04)

Unique identifier for health care providers: H for any health care provider

Tool WP3: 3/2 (part 2 of 2)

Country	□□
Hospital	□□
Tool	□/□
Type	H
Participant	####
□ □/□ □/□/□/H/##	

Observer: Name (capital letters) ___________________ **Signature____________________ Date: ___/___/**

Supervisor: Name (capital letters) __________________ **Signature____________________ Date: ___/___/**

Data entry personnel: Name (capital letters) ___________________**Signature________________Date: ___/___/**

Operational definition:

Done: Performs the step or task according to the standard procedure or guidelines.
Not done: Unable to perform the step or task according to the standard procedure or guidelines.
Not applicable: Step or task not applicable for Mary during evaluation by observer.

PERFORMANCE STANDARDS	No	Variable name	VERIFICATION CRITERIA	Observation Done=1, Not done=0, Not applicable=9	COMMENTS
**Role play 1_admission**					
Section 1. Admission<br><br>***Scenario 1: Mary is a 28-year-old para 2***<br><br>***She is 38+6 weeks pregnant and has just presented at your health care facility with a history of contractions for the past 2 hours. Please perform an initial assessment of Mary.***	1	s1q1	When assessing Mary, the provider;<br>determines if active labour has started (fetal wellbeing, perspiration, anxiety)		
	Asks Mary whether she has or had:				
	2	s1q2	Vaginal bleeding		
	3	s1q3	Loss of fluid from vagina		
	4	s1q4	Convulsions		
	5	s1q5	Severe headache and blurred vision		
	6	s1q6	Severe abdominal pain		
	7	s1q7	Respiratory difficulty		
	8	s1q8	Fever		
	9	s1q9	Records the information on Mary's clinical history		
	10	s1q10	Refers Mary after providing immediate treatment if required		

PERFORMANCE STANDARDS	No	Variable name	VERIFICATION CRITERIA	Observation Done=1, Not done=0, Not applicable=9	COMMENTS
**Section 2. The provider treats Mary in a cordial manner.**	1	s2q1	Greets Mary and her husband or companion in a cordial manner and introduces her/himself		
	2	s2q2	Ensures that she/he speaks in easy-to-understand lay language (does not use technical words) with Mary		
	3	s2q3	Explains to Mary and her companion the assessment procedures and asks for consent to perform them		
	4	s2q4	Encourages Mary and her companion to ask questions		
	5	s2q5	Listens to what the Mary and her companion have to say		
	6	s2q6	Responds to questions and concerns		
	7	s2q7	Provides emotional support and reassurance		
	8	s2q8	Responds to Mary's immediate needs (thirst, hunger, cold/hot, need to urinate, etc.)		
**Section 3. The provider properly reviews and fills out the clinical history about Mary.**	1	s3q1	Asks and records Mary's clinical history following information in a confidential and private environment and in a non-judgemental way		
	2	s3q2	Age of Mary [in this skill drill it is 28 years]		
	Previous obstetric history:				
	3	s3q3	Number of pregnancies		
	4	s3q4	Number of abortions		
	5	s3q5	Number of normal births		
	6	s3q6	Number of caesarean sections		

PERFORMANCE STANDARDS	No	Variable name	VERIFICATION CRITERIA	Observation Done=1, Not done=0, Not applicable=9	COMMENTS
	7	s3q7	Number of children born alive and still alive		
	8	s3q8	Number of still births		
	9	s3q9	Any maternal complication in any previous pregnancy		
	10	s3q10	Date and outcome of last pregnancy		
	Other general medical problems (for diabetes, hypertension, asthma etc.):				
	11	s3q11	Any medication		
	12	s3q12	Use of alternative medications or herbs		
	13	s3q13	Receiving treatment for tuberculosis and/or HIV		
	Gestational age – ask or calculate:				
	14	s3q14	Last menstrual period (LMP)		
	15	s3q15	Expected date of delivery (EDD)		
	Contractions				
	16	s3q16	Avoids asking questions during contractions		
	17	s3q17	Asks Mary about when her labour/when the painful regular contractions began		
	18	s3q18	Frequency and strength of the contractions		
	Asks about Mary's "bag of waters"/membrane				
	19	s3q19	When the water broke?		
	20	s3q20	What colour the water had?		

PERFORMANCE STANDARDS	No	Variable name	VERIFICATION CRITERIA	Observation Done=1, Not done=0, Not applicable=9	COMMENTS
	21	s3q21	What smell the water had?		
	22	s3q22	Whether Mary feels the baby's movements		
	23	s3q23	Records the information on clinical history form		
**Section 4. The provider properly conducts the physical examination between contractions and if time allows.**	1	s4q1	Helps Mary onto the examination table/bed		
	Ensures privacy during the entire process of provision of care:				
	2	s4q2	Ensures privacy with a screen or curtain to separate the woman from others, at least during examination		
	3	s4q3	Ensures that Mary remains covered with her robe or clothing		
	4	s4q4	Asks Mary to urinate (tests urine for albumin and sugar)		
	5	s4q5	Washes hands		
	6	s4q6	Explains each step of the examination to Mary using easy-to-understand language		
	Takes or delegates vital signs to assistant:				
	7	s4q7	Temperature		
	8	s4q8	Blood pressure (BP)		
	9	s4q9	Pulse		
	10	s4q10	Respiratory rate		
	11	s4q11	Oedema		
	12	s4q12	Checks the conjunctiva for anemia		

PERFORMANCE STANDARDS	No	Variable name	VERIFICATION CRITERIA	Observation Done=1, Not done=0, Not applicable=9	COMMENTS
	13	s4q13	Tests blood for haemoglobin if anemia suspected		
	14	s4q14	Observes Mary's emotional response to labour		
**Section 5. The provider properly conducts the obstetric examination between contractions.**	1	s5q1	Observes the shape and size of the abdomen and checks for the presence of scars		
	2	s5q2	Determines fundal height, fetal lie and presentation, engagement of presenting part		
	3	s5q3	Evaluates uterine contractions (frequency, strength and duration over a 10-minute period)		
	4	s5q4	Auscultates fetal heart rate (FHR)		
	5	s5q5	Records the results of the obstetric examination on the clinical history form		
**Section 6. The provider properly conducts a vaginal examination.**	1	s6q1	Puts on a clean plastic or rubber apron		
	2	s6q2	Performs hand hygiene and puts sterile/HLD gloves on both hands		
	3	s6q3	Asks for permission to perform a vaginal examination. Examines the vulva (ulcers, blood, liquid, secretion, presenting part)		
	4	s6q4	Cleanses the perineum with nonalcoholic antiseptic solution using the hand that will not be used to perform the examination		
	Performs per vaginal examination following standard technique:				
	5	s6q5	Gently inserts two lubricated fingers of the examining hand into the vagina		
	6	s6q6	Notes the length, texture and dilatation of the cervix		

PERFORMANCE STANDARDS	No	Variable name	VERIFICATION CRITERIA	Observation Done=1, Not done=0, Not applicable=9	COMMENTS
	7	s6q7	Notes if the membranes are intact, or if they have ruptured, ensures the cord has not prolapsed		
	8	s6q8	Measures the level of the presenting part in centimeters above or below Mary's ischial spines		
	9	s6q9	Identifies fetal presentation and determines fetal position by palpating the features of the presenting part (e.g. fontanelles in cephalic presentation)		
	10	s6q10	If gloves are disposable, places them in a waste-container with a plastic liner; OR<br>if they are reusable, immerses them in a 0.5% chlorine solution for at least 10 minutes before transferring them for sterilization		
	11	s6q11	Performs hand hygiene after removing gloves		
	12	s6q12	Record the results of the vaginal examination on the clinical history form		
**Section 7. Documentation**	1	s7q1	Record Mary's information and assessment of findings		

PERFORMANCE STANDARDS	No	Variable name	VERIFICATION CRITERIA	Observation Done=1, Not done=0, Not applicable=9	COMMENTS
**Role play 2_childbirth and 15 minutes postpartum**					
Scenario 2: Mary's labour has progressed normally, and she is now feeling rectal pressure and an urge to push. Assume that Mary is fully dilated.<br><br>***Please demonstrate the care that you would provide for Mary from this point until 15 minutes following the birth of the baby.***<br><br>**Section 8. The provider assists the woman to have a safe and clean birth.**	1	s8q1	Preparation of equipment (e.g. delivery kit, oxytocin, gloves etc.). Washes hands thoroughly with soap and water and dries them		
	2	s8q2	Puts sterile or HLD gloves on both hands		
	3	s8q3	Puts on personal protective equipment (plastic/rubber apron)		
	4	s8q4	Cleanses the perineum with water or a nonalcoholic antiseptic solution after asking for permission to do so		
	5	s8q5	Monitors, or has an assistant monitor, FHR every five minutes during second stage		
	6	s8q6	Supports Mary to bear down when she feels the desire in the position she chooses (does not force her to bear down)		
	7	s8q7	Performs an episiotomy only if necessary (breech, shoulder dystocia, forceps, vacuum, poorly healed 3rd or 4th degree tear, or fetal distress) as the perineum distends		
	8	s8q8	Administers local anaesthesia		
	9	s8q9	Waits to perform episiotomy until the perineum is thinned out and the baby's head is visible during a contraction		
	10	s8q10	If delivery of the head does not follow immediately, applies pressure to the episiotomy site between contractions to minimize bleeding		

PERFORMANCE STANDARDS	No	Variable name	VERIFICATION CRITERIA	Observation Done=1, Not done=0, Not applicable=9	COMMENTS
	11	s8q11	Allows the head to spontaneously crown while guarding the perineum and episiotomy (if given)		
	12	s8q12	After the emergence of the head, asks Mary to briefly refrain from bearing down (open mouth breathing) and explains how to perform open mouth breathing		
	13	s8q13	After fetal head is born, allows the neck to gradually extend and quickly palpates to determine if cord around the neck;<br>- if it is loose, slides it over the baby's head;<br>- if it is very tight, clamps it in two places and cuts it before unravelling it from around the baby's neck		
	14	s8q14	Assists Mary in birthing the baby - allows spontaneous restitution and external rotation of the head without manipulation		
	15	s8q15	Guides the baby's head and chest in an upward curve until the posterior shoulder has emerged over the perineum		
	16	s8q16	Holds the baby around the chest to aid the birth of the trunk and lift it towards Mary's abdomen		
	17	s8q17	Places the baby on a dry towel/cloth on Mary's abdomen		
	18	s8q18	Cleans the baby's mouth and nose using a sterile gauze if meconium present		
	19	s8q19	Wipes the baby's eyes with a clean piece of cloth		
	20	s8q20	Clamps and cuts the cord using clean/sterile blade/ instrument within 1-3 minutes of the birth and cut it close to the perineum		

PERFORMANCE STANDARDS	No	Variable name	VERIFICATION CRITERIA	Observation Done=1, Not done=0, Not applicable=9	COMMENTS
	21	s8q21	Informs Mary of the sex of her child (with same enthusiasm if male or female)		
	22	s8q22	Assists Mary to move her child up to her chest for skin-to-skin contact		
**Section 9. The provider properly conducts a rapid initial assessment and provides immediate newborn resuscitation if needed.**	1	s9q1	Receives the baby either by a different provider with separate gloves or by same provider using a new set of gloves		
	2	s9q2	Dries the baby with a clean dry towel from head to feet		
	3	s9q3	Discards the used towel and covers the baby including the head with a clean dry towel		
	4	s9q4	Determines whether the baby is breathing		
	If the baby does not begin breathing or is breathing with difficulty				
	5	s9q5	Asks assistance		
	If the baby breathes from birth				
	6	s9q6	Facilitates delayed cord clamping (allows 1-3 minutes before clamping and cutting the cord).		
	7	s9q7	If the baby is breathing normally, place the baby in skin-to-skin contact on Mary's chest or abdomen		
	8	s9q8	Encourages "Baby Crawl" practice and immediate breastfeeding		
	9	s9q9	Notes the date and time of birth		
	10	s9q10	Informs the attendant of the baby's condition		

PERFORMANCE STANDARDS	No	Variable name	VERIFICATION CRITERIA	Observation Done=1, Not done=0, Not applicable=9	COMMENTS
**Section 10. The provider adequately performs active management of the third stage of labour.**	1	s10q1	Touches Mary's abdomen to rule out the presence of a second baby (without stimulating contractions)		
	2	s10q2	Give inj. Oxytocin 10 units direct I/M and wait for next contraction within one minute after birth of the baby.		
	3	s10q3	Holds the cord along with clamp and provides controlled cord traction/sustained downward traction		
	4	s10q4	Repeats controlled cord traction while simultaneously applying counter pressure above pubis to guard uterus (places the other hand above the level of the symphysis pubis with hand facing towards the umbilicus to provide counter traction on the uterus)		
	5	s10q5	Applies steady tension by pulling the cord firmly and maintaining pressure (jerky movements and force must be avoided)		
	6	s10q6	When the placenta is visible at the vulva holds the placenta with both hands, assists in the expulsion of the placenta, by turning it over in the hands, without applying traction,		
	7	s10q7	Uses a gentle and upward and downward movement or twisting action to deliver the membranes, "teasing out" the membranes		
	8	s10q8	Checks whether the uterus is well contracted		
	9	s10q9	Massages the uterus with one hand on a cloth over the abdomen, until the uterus contracts firmly		
	10	s10q10	Checks the placenta on a clean table or the delivery bed with a good light source: whether all the lobules are present and fit together to see if		

PERFORMANCE STANDARDS	No	Variable name	VERIFICATION CRITERIA	Observation Done=1, Not done=0, Not applicable=9	COMMENTS
			complete (missing cotyledon) and to identify any abnormalities such as aberrant vessels or nods in the umbilical cord		
	11	s10q11	Inspects the membranes for completeness		
	12	s10q12	Notes the position of insertion of the cord		
	13	s10q13	Inspect the cut end of the cord for the presence of two arteries and one vein		
**Section 11. The provider adequately performs immediate postpartum care.**	1	s11q1	Ensures direct strong light into the perineum, wearing gloves, that Mary is in a comfortable position, and swabs are ready		
	2	s11q2	Informs and gains consent from Mary to check her perineum		
	3	s11q3	Inspects the lower vagina and perineum for lacerations/tear and checks for increased bleeding from the cervix/cervical tear		
	4	s11q4	Gently cleanses the vulva and perineum with clean water or a nonalcoholic antiseptic solution and dries with a clean, soft cloth		
	5	s11q5	Sutures tears/episiotomy, if necessary		
	6	s11q6	Makes sure that Mary is comfortable (clean, hydrated and warmly covered)		
	**Section 12. Documentation of AMTSL**				
	1	s12q1	Performs hand hygiene after removing gloves		
	2	s12q2	Records relevant details (childbirth, active management of the third stage of Labour (AMTSL), placenta examination, newborn condition, episiotomy (if done) on the Mary's record		

Anything from the role play/observations you would like to tell us?
